# Medium-term oncological outcomes of totally laparoscopic colectomy with intracorporeal anastomosis for right-sided and left-sided colon cancer: propensity score matching analysis

**DOI:** 10.1186/s12893-022-01798-3

**Published:** 2022-09-19

**Authors:** Hiroki Hamamoto, Yusuke Suzuki, Yoshiaki Takano, Toru Kuramoto, Masatsugu Ishii, Wataru Osumi, Shinsuke Masubuchi, Keitaro Tanaka, Kazuhisa Uchiyama

**Affiliations:** Department of General and Gastroenterological Surgery, Osaka Medical and Pharmaceutical University, 2-7 Daigaku-Machi, Takatsuki, 569-8686 Japan

**Keywords:** Totally laparoscopic colectomy, Intracorporeal anastomosis, Propensity score matching analysis

## Abstract

**Background:**

This retrospective study aimed to compare long-term oncological outcomes between laparoscopic-assisted colectomy (LAC) with extracorporeal anastomosis (EA) and totally laparoscopic colectomy (TLC) with intracorporeal anastomosis (IA) for colon cancers, including right- and left-sided colon cancers.

**Methods:**

Patients with stage I–III colon cancers who underwent elective laparoscopic colectomy between January 2013 and December 2017 were analyzed retrospectively. Patients converted from laparoscopic to open surgery and R1/R2 resection were excluded. Propensity score matching (PSM) analysis (1:1) was performed to overcome patient selection bias.

**Results:**

A total of 388 patients were reviewed. After PSM, 83 patients in the EA group and 83 patients in the IA group were compared. Median follow-up was 56.5 months in the EA group and 55.5 months in the IA group. Estimated 3-year overall survival (OS) did not differ significantly between the EA group (86.6%; 95% confidence interval (CI), 77.4–92.4%) and IA group (84.8%; 95%CI, 75.0–91.1%; *P* = 0.68). Estimated 3-year disease-free survival (DFS) likewise did not differ significantly between the EA group (76.4%; 95%CI, 65.9–84.4%) and IA group (81.0%; 95%CI, 70.1–88.2%; *P* = 0.12).

**Conclusion:**

TLC with IA was comparable to LAC with EA in terms of 3-year OS and DFS. TLC with IA thus appears to offer an oncologically feasible procedure.

## Introduction

The advantages of totally laparoscopic colectomy (TLC) with intracorporeal anastomosis (IA) for right-sided colon cancer (RC) have been reported in many studies [1–7]. TLC with IA can avoid the risk of twisting the mesentery and bowel during anastomotic construction and allows the surgeon to select the optimal site for bowel extraction. A systematic review [6] confirmed better short-term outcomes of TLC with IA for RC compared with laparoscopic-assisted colectomy (LAC) with extracorporeal anastomosis (EA) in terms of results such as bowel function, length of hospital stay, and cosmetic results. In addition, Hanna et al. [8] showed no significant difference between LAC with EA and TLC with IA for RC in terms of oncological outcomes.

IA for sigmoid colon and rectal cancer using a double-stapling technique (DST) with a circular stapler is a common procedure. If the tumor is located in the descending colon or proximal sigmoid colon where DST anastomosis is difficult, EA such as functional end-to-end or side-to-side colo-colostomy is usually performed. LAC with EA for left-sided colon cancer (LC) needs mobilization of the splenic flexure, whereas TLC with IA might omit that procedure. We have reported that TLC with IA is technically feasible for LC where DST anastomosis is difficult and can be performed with good cosmetic outcomes and decreased time to first flatus [9]. Swaid et al. [10] reported similar results, but few studies have compared TLC with IA to LAC with EA for LC. Moreover, no reports have been published on the long-term outcomes of TLC with IA compared to LAC with EA for colon cancers, including RC and LC.

The present study aimed to compare long-term outcomes between LAC with EA and TLC with IA for colon cancers including LC, using propensity score matching (PSM) analysis to reduce sample selection bias.

## Materials and methods

We started TLC with IA from June 2013 for patients with early-stage colon cancer, and the indications were gradually and carefully expanded to include advanced cancer. Patients who underwent elective laparoscopic surgery for colon cancer in our hospital between January 2013 and December 2017 were retrospectively evaluated. The study protocol was approved by the Institutional Review Board of Osaka Medical and Pharmaceutical University (approval date: January 24, 2020, chairman: Junko Tamaki, acceptance number: 2853) and was performed in accordance with the Declaration of Helsinki. Due to the retrospective design of the study, the local ethic committee of Osaka Medical and Pharmaceutical University confirmed that informed consent was not necessary from participants. The choice of operation, as either LAC with EA or TLC with IA, was determined by the operating surgeon. The standard procedures were similar for all patients and performed by the same surgical team. Exclusion criteria were: DST anastomosis; patients with distant metastasis or simultaneous double cancer; conversion from laparoscopic to open surgery; or R1/R2 resection.

### Operative procedure

Patients were placed in the lithotomy position with a 0–15° head-up or head-down tilt during surgery. Intraabdominal pressure was maintained at 10 mmHg, and pneumoperitoneum was established with heated, humidified carbon dioxide gas. Five trocars were used in all procedures, with mobilization of the colon and lymphadenectomy performed laparoscopically by the medial-to-lateral approach. No drainage tube was placed after surgery.

### LAC with EA

**Fig. 1:**
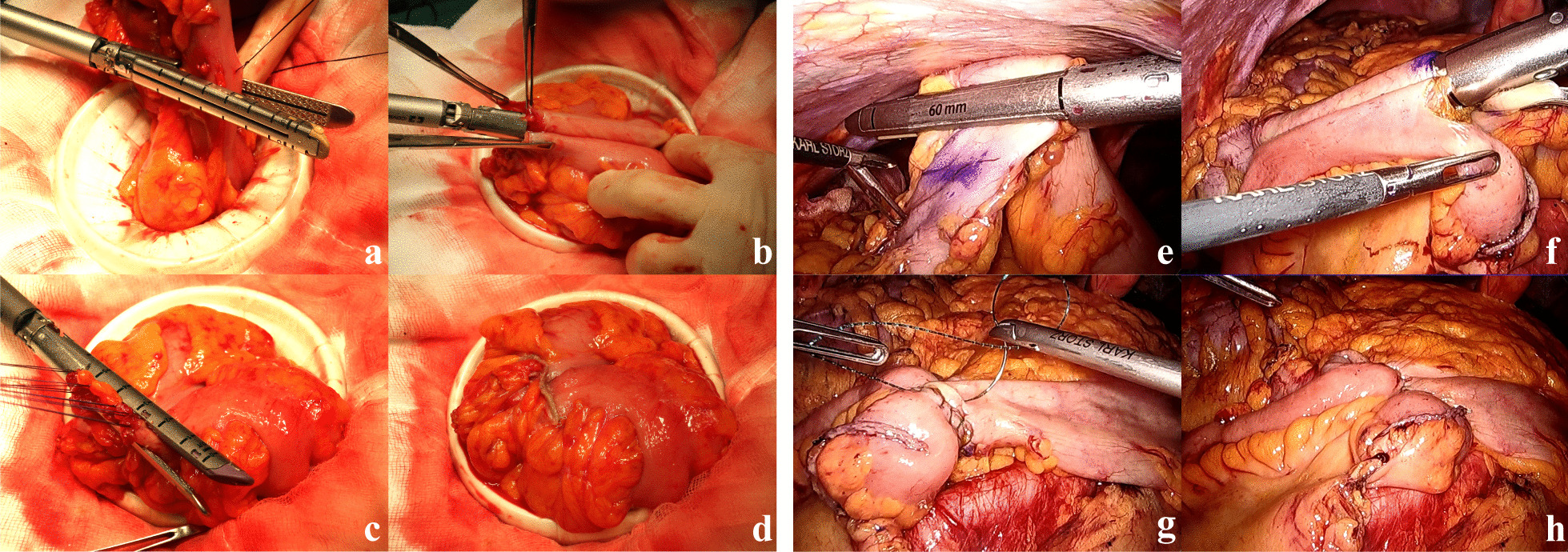
**a–d** Extracorporeal anastomosis. **e–h** Intracorporeal anastomosis

A small incision was extended for the trocar incision at the umbilicus or the left lower quadrant port site. After protecting with Wound Protector, the bowel was externalized. The ileum or colon was resected with 60-mm linear staples (Fig. 1a) and ileo-colostomy/colo-colostomy was performed using a 60-mm linear stapler (Fig. 1b). The enterotomy was then closed using the 60-mm linear stapler (Fig. 1c, d).

### TLC with IA

The procedure for TLC with IA for RC or LC has been described in our previous reports [9, 11]. In summary, the ileum or colon was divided intracorporeally with 60-mm linear staplers (Fig. 1e), and the enterotomy was closed using the Albert-Lembert method after fashioning side-to-side ileo-colostomy/colo-colostomy with a 60-mm linear stapler (Fig. 1f–h). The specimen was then extracted through a mini-laparotomy over the trans-umbilical port site, the lower quadrant port site, or via Pfannenstiel laparotomy.

### Surveillance after surgery

Surveillance after surgery was performed in accordance with Japanese guideline. In short, the patients received interviews, physical examinations, tumor marker examinations, and whole-body computed tomography every 6 months.

### Statistical analysis

Statistical analysis was performed using JMP 14.2 software (SAS Institute Inc., Cary, NC, USA). To minimize the influence of potential confounders on selection bias, propensity scores of patient characteristics and pathological results were generated using binary logistic regression. One-to-one matching between two groups was accomplished using the nearest-neighbor matching method, performed without replacement, and using a caliper width of 0.2 standard deviations of the logit of the estimated propensity score. After propensity score matching, the two matched groups were handled as unpaired independent groups. Data are expressed as median with interquartile range or mean ± standard error. The statistical significance of other data was determined using a one-way analysis of variance, Fisher’s test, the chi-squared test, or Student’s t test. Univariate analyses of overall survival (OS) and disease-free survival (DFS) rates were performed using the Kaplan-Meyer method. Differences when comparing survival curves were analyzed using the log-rank test. Cox regression analysis was performed to identify possible prognostic factors. Results are reported as hazard ratios and 95% confidence intervals (CIs). Values of *P* < 0.05 were considered statistically significant.

## Results

### Patients

**Fig. 2:**
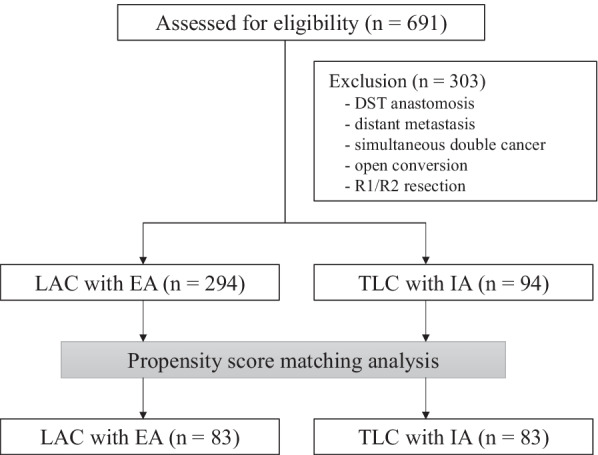
Flow diagram of this study. *DST* double stapling technique, *LAC* laparoscopic-assisted colectomy, *EA* extracorporeal anastomosis, *TLC* totally laparoscopic colectomy, *IA* intracorporeal anastomosis

Of the 691 eligible patients, 303 patients were excluded, and 388 patients were analyzed in this study (Fig. 2). We identified significant differences in age, body mass index, American Society of Anesthesiologists score, tumor location, and PSM was performed. Patient background characteristics and pathological results in both groups were closely balanced by the PSM, resulting in 83 matched pairs (Table 1). Finally, 83 patients in the EA group and 83 patients in the IA group were compared.

**Table 1:** Patient characteristics and pathological results before and after PSM

		Before PSM				After PSM		
	EA (n = 294)	IA (n = 94)	*P *value		EA (n = 83)		IA (n = 83)	*P *value
Sex (M/F)	135/159	54/40	0.051		39/44		48/35	0.16
Age (years)	73 (29–90)	70 (29–87)	0.036*		70 (34–88)		71 (29–87)	0.92
BMI (kg/m^2^)	22 (13–33)	23 (15–41)	< 0.01*		23 (17–33)		23 (15–41)	0.48
ASA classification (I/II/III)	70/187/37	52/39/3	< 0.01*		42/39/2		41/39/3	0.90
History of diabetes	37 (13%)	10 (11%)	0.61		10 (12%)		10 (12%)	1.00
Tumor location			< 0.01*					0.74
Right-side colon	234 (80%)	52 (55%)			53 (64%)		51 (61%)	
Left-side colon	60 (20%)	42 (45%)			30 (36%)		32 (39%)	
Tumor size			0.41					0.32
> 5 cm	93 (32%)	34 (36%)			24 (29%)		30 (36%)	
≤ 5 cm	201 (68%)	60 (64%)			59 (71%)		53 (64%)	
T stage			0.081					0.07
T1–2	120 (41%)	29 (31%)			35 (42%)		24 (29%)	
T3–4	174 (59%)	65 (69%)			48 (58%)		59 (71%)	
LN metastasis			0.21					0.29
Yes	71 (24%)	29 (31%)			19 (23%)		25 (30%)	
No	223 (76%)	65 (69%)			64 (77%)		58 (70%)	
TNM staging			0.067					0.23
I	111 (38%)	26 (28%)			32 (39%)		22 (27%)	
II	112 (38%)	39 (42%)			32 (39%)		36 (43%)	
III	71 (24%)	29 (30%)			19 (23%)		25 (30%)	

*PSM* propensity score matching, *EA* extracorporeal anastomosis, *IA* intracorporeal anastomosis, *M* male, *F* female, *BMI* body mass index, *ASA* American Society of Anesthesiologists, *LN* lymph-node

TNM stage is classified by UICC-7 staging. Values are expressed as median (range)

^*^Significant difference between groups; *P* < 0.05

### Operative, pathological, and oncological results after PSM

Operative, pathological, and oncological results before and after PSM are shown in Table 2. We identified significant differences in distal resection margin, operation time, length of skin incision, and surgical site infection (SSI) rate. Time to first flatus, length of hospital stay, and leakage rate did not differ significantly. Two patients (2.4%) in the EA group and three patients (3.6%) in the IA group experienced disseminated recurrence, but no patients experienced anastomosis site recurrence after PSM.

**Table 2:** Operative, pathological, and oncological results

			Before PSM				After PSM		
	EA (n = 294)	IA (n = 94)		P value		EA (n = 83)		IA (n = 83)	*P* value
Operation time (min)	190 (79–528)	227 (115–530)		< 0.01*		200 (110–372)		227 (85–530)	< 0.01*
Blood loss (ml)	10 (10–50)	10 (10–220)		0.27		10 (10–440)		10 (10–100)	0.44
Number of harvested lymph nodes	18 (0–74)	18 (0–54)		0.53		18 (1–48)		19 (0–54)	0.93
Resection margin									
Proximal (cm)	10 (3–40)	10 (2–35)		0.49		10 (4–30)		10 (2–35)	0.69
Distal (cm)	10 (0.5–35)	10 (0.5–30)		0.021*		9 (0.5–20)		10 (0.5–30)	0.015*
Skin incision (cm)	4.5 ± 1.7	4.0 ± 1.1		< 0.01*		4.6 ± 0.2		4.1 ± 0.2	< 0.01*
Time to first flatus (days)	2 (0–8)	1 (0–4)		0.079		2 (0–4)		1 (0–4)	0.38
Length of hospital stay (days)	11 (7–154)	12 (7–87)		0.35		11 (7–56)		12 (7–87)	0.025*
Complications									
Superficial SSI	19 (10%)	13 (19%)		0.030*		3 (3.6%)		11 (13%)	0.047*
Organ space/deep SSI	11 (3.7%)	5 (5.3%)		0.50		4 (4.8%)		5 (6.0%)	0.73
Leakage	7 (2.4%)	6 (6.4%)		0.092		2 (2.4%)		5 (6.0%)	0.23
≥ Clavien-Dindo III	12 (4.1%)	9 (9.6%)		0.063		4 (4.8%)		9 (11%)	0.14
Recurrence									
Liver/lung	19 (6.5%)	7 (7.5%)		0.74		2 (2.4%)		7 (8.4%)	0.087
Dissemination	9 (3.1%)	3 (3.2%)		0.95		2 (2.4%)		3 (3.6%)	0.65
Anastomosis site	2 (0.7%)	1 (1.1%)		0.71		0		0	

*PSM* propensity score matching, *EA* extracorporeal anastomosis, *IA* intracorporeal anastomosis, *SSI* surgical site infection

Values are expressed as median (range) or average ± SD

^*^Significant difference between groups; *P* < 0.05

### Medium-term oncological outcomes after PSM

**Fig. 3:**
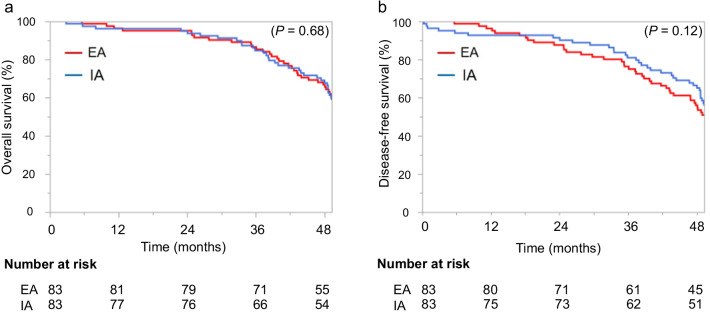
Kaplan–Meier curves comparing overall survival (**a**) and disease-free survival (**b**) in totally laparoscopic colectomy between intracorporeal anastomosis (IA) and extracorporeal anastomosis (EA)

Median follow-up was 56.5 months for the EA group and 55.5 months for the IA group. At the time of the last follow-up on December 31, 2020, 10 patients had died, comprising 4 patients (4.8%) from the EA group and 6 patients (7.2%) from the IA group. Estimated 3-year OS did not differ significantly between the EA group (86.6%; 95%CI, 77.4–92.4%) and the IA group (84.8%; 95%CI, 75.0–91.1%; *P* = 0.68) (Fig. 3a). Estimated 3-year DFS likewise did not differ significantly between the EA group (76.4%; 95%CI, 65.9–84.4%) and the IA group (81.0%; 95%CI, 70.1–88.2%; *P* = 0.12) (Fig. 3b).

### Cox regression analysis after PSM

Neither EA nor IA was associated with poor DFS (Table 3). Tumor location and lymph-node metastasis were likewise not associated with poorer DFS. No independent risk factors were identified.

**Table 3:** Cox regression analysis for possible prognostic factors after PSM

	Hazard ratio	95%CI	*P* value
Sex			
Male	1		
Female	0.86	0.64–1.18	0.35
Age (years)			
≤ 65	1		
> 65	1.04	0.75–1.50	0.82
BMI (kg/m^2^)			
≤ 25	1		
> 25	1.32	0.92–1.90	0.13
Tumor location			
Right-side colon	1		
Left-side colon	1.22	0.89–1.68	0.21
Anastomosis			
Extracorporeal	1		
Intracorporeal	0.80	0.59–1.10	0.80
T stage			
T1–2	1		
T3–4	1.14	0.82–1.58	0.42
LN metastasis			
No	1		
Yes	1.23	0.87–1.74	0.24
Adjuvant chemotherapy			
No	1		
Yes	1.13	0.76–1.68	0.55

*PSM* propensity score matching, *CI* confidence interval, *BMI* body mass index, *LN* lymph-node

TNM stage is classified by UICC-7 staging

## Discussion

Our results showed that laparoscopic colectomy with TLC with IA was similar to LAC with EA in terms of 3-year OS and DFS. IA involved exposing the ileum and colon under pneumoperitoneum in the abdominal cavity, raising concerns of increased peritoneal dissemination with IA. In the 1990s, when laparoscopic surgery for colon cancer was started, concern about port-site recurrences was widespread but was allayed by large prospective series [12–15]. In the present study including TLC with IA for LC, only 2 patients in the EA group and 3 patients in the IA group experienced disseminated recurrence after PSM. No patients in either group experienced recurrence at the anastomosis site after PSM. Moreover, neither EA nor IA was associated with poor DFS in Cox regression analysis. No significant differences in the number of lymph nodes harvested were identified between groups. These results suggested that TLC with IA is an oncologically reasonable procedure. This cohort before PSM included 1 patient in the IA group and 1 patient in the EA group without lymph node harvested. Although it was not sufficient for colectomy, both patients were included in this cohort and no recurrence had been observed in them.

In this cohort after PSM, median harvested lymph nodes were 18 in the EA group and 19 in the IA group, which might be small. This result was due to the Japanese so-called 10 cm rule. Previously, some studies have shown that longitudinal spread greater than 10 cm beyond the tumor was extremely rare, at 1–4% for right-sided tumors and 0% for left-sided tumors [16, 17]. For this reason, in Japan, most specimens were not greater than 10 cm beyond the tumor.

In this study, the incidence of superficial SSI was significantly higher in the IA group (13%) than in the EA group (3.6%; *P* = 0.047). Exposing the intestinal tract in the abdominal cavity during IA procedures may result in exposure to bacteria, but the frequency of organ-space/deep SSI did not differ significantly between groups in our results (4.8% in the EA group vs. 6.0% in the IA group, *P* = 0.73). We showed that IA procedures did not increase the risk of dissemination, but also did not increase organ-space/deep SSI. Superficial SSI was frequently encountered at the port site through which 60-mm linear staplers were passed for side-to-side ileo-colostomy/colo-colostomy. The port through which the linear stapler was passed for anastomosis may become contaminated with stool, increasing the risk of superficial SSI. Length of postoperative hospital stay is known to be prolonged by the occurrence of SSI [18], and the IA group showed a longer hospital stay than the EA group in our study. Several reports showing that TLC with IA was favorable in terms of SSI have represented the rate of SSI as 1–4.4% [19–21], lower than our results. To prevent SSI, we have started applying additional steps, such as cleaning inside the contaminated port and administering chemical preparation with oral antibiotics the day before surgery. Since introducing such steps, we have encountered no SSIs in 13 consecutive TLCs with IA for RC in 2020 [11].

Good short-term outcomes of TLC with IA for RC have been shown in many retrospective reports [1–7]. In this study including LC, TLC with IA required a longer operation time and needed a shorter skin incision than LAC with EA. Because of the procedure dissecting the mesentery and anastomosis intracorporeally, TLC with IA took longer, but the smaller skin incision was advantageous in terms of cosmetology and postoperative pain. As another advantage, TLC with IA enables extraction site flexibility [19]. This advantage is particularly useful in natural orifice specimen extraction surgery [22] and holds promise for scarless surgery if natural orifice transluminal endoscopic surgery can be achieved.

The potential advantage of IA lies in surgeries for patients who have undergone poly-surgery. Patients who have undergone polysurgery might also encounter difficulties with EA due to intra-abdominal adhesions, in which cases IA would be useful.

Adequate proximal and distal margins are important to guarantee sufficient oncological radicality. The IA group obtained a significantly longer distal margin in this study. TLC with IA, which determined the dissection line in the abdominal cavity, can secure a wider disease-free margin compared to EA. Scatizzi et al. [4] reported similar results, with resection of a longer specimen potentially reducing the risk of residual colon ischemia.

The present study showed that none of the tumor locations, lymph-node metastasis, or stage III were associated with poor DFS. Two large population-based studies have reported that RCs show a worse prognosis than left-sided colon cancers [23, 24], but tumor location was not a prognostic factor in our study. Similarly, lymph-node metastasis and stage III are considered to be associated with poor DFS, but again, not in our study. The benefit of adjuvant chemotherapy (AC) for stage III colon cancer has been clearly established [25, 26], and AC is recommended for patients with lymph-node metastasis; in other words, patients classified as stage III according to National Comprehensive Cancer Network guidelines [27]. The 3-year OS rate in this study including stage III cases (166 patients after PSM) was 85.7%. Our overall prognosis in this study might have been too good and the sample size too small to allow the identification of significant prognostic factors.

To the best of our knowledge, two prospective studies have compared TLC with IA to LAC with EA. Marco et al. [28] conducted a prospective, randomized study of 140 patients with RC, reporting the earlier recovery of postoperative bowel function for TLC with IA compared to LAC with EA, but they did not meet their primary endpoint of shorter hospital stay. Similarly, Bollo et al. [29] conducted a prospective, randomized study of 140 patients with RC, and did not meet the primary endpoint of shorter hospital stay in the IA group compared to the EA group. Serra-Aracil et al. [30] are carrying out a prospective, controlled, nonrandomized study, the HEMI-D-TREND Study, in which the primary endpoint is overall morbidity and mortality for LAC with EA and TLC with IA, and open colectomy for RC. The study is ongoing and expected to finish in June 2021, and the results are eagerly awaited. No prospective studies including both RC and LC have been reported yet.

This study had some limitations, including the retrospective, single-center design, small sample size, and underpowered. However, strengths of the study include the fact that this is the largest retrospective comparison of long-term, oncological outcomes between LAC with EA and TLC with IA, the reduction of selection bias by PSM, and the inclusion of not only RC but also LC.

## Conclusions

The present study showed that TLC with IA was an oncologically feasible procedure with long-term outcomes comparable to those of LAC with EA. A prospective, randomized trial comparing LAC with EA to TLC with IC could validate these findings.

## Data Availability

The datasets generated and analyzed during the current study available from the corresponding author on reasonable request.

## Abbreviations

LAC: Laparoscopic-assisted colectomy
EA: Extracorporeal anastomosis
TLC: Totally laparoscopic colectomy
IA: Intracorporeal anastomosis
PSM: Propensity score matching
OS: Overall survival
CI: Confidence interval
DFS: Disease-free survival
RC: Right-sided colon cancer
DST: Double-stapling technique
SSI: Surgical site infection

## References

[1] Raftopoulos I, Courcoulas AP, Blumberg D (2006). Should completely intracorporeal anastomosis be considered in obese patients who undergo laparoscopic colectomy for benign or malignant disease of the colon?. Surgery.

[2] Bergamaschi R, Schochet E, Haughn C, Burke M, Reed JF, Arnaud JP (2008). Standardized laparoscopic intracorporeal right colectomy for cancer: short-term outcome in 111 unselected patients. Dis Colon Rectum.

[3] Grams J, Tong W, Greenstein AJ, Salky B (2010). Comparison of intracorporeal versus extracorporeal anastomosis in laparoscopic-assisted hemicolectomy. Surg Endosc.

[4] Scatizzi M, Kröning KC, Borrelli A, Andan G, Lenzi E, Feroci F (2010). Extracorporeal versus intracorporeal anastomosis after laparoscopic right colectomy for cancer: a case–control study. World J Surg.

[5] Anania G, Santini M, Scagliarini L, Marzetti A, Vedana L, Marino S, Gregorio C, Resta G, Cavallesco G (2012). A totally mini-invasive approach for colorectal laparoscopic surgery. World J Gastroenterol.

[6] Cirocchi R, Trastulli S, Farinella E, Guarino S, Desiderio J, Boselli C, Parisi A, Noya G, Slim K (2013). Intracorporeal versus extracorporeal anastomosis during laparoscopic right hemicolectomy—systematic review and meta-analysis. Surg Oncol.

[7] Milone M, Elmore U, DiSalvo E, Delrio P, Bucci L, Ferulano GP, Napolitano C, Angiolini MR, Bracale U, Clemente M (2015). Intracorporeal versus extracorporeal anastomosis. Results from a multicentre comparative study on 512 right-sided colorectal cancers. Surg Endosc..

[8] Hanna MH, Hwang GS, Phelan MJ, Bui TL, Carmichael JC, Mills SD, Stamos MJ, Pigazzi A (2016). Laparoscopic right hemicolectomy: short- and long-term outcomes of intracorporeal versus extracorporeal anastomosis. Surg Endosc.

[9] Masubuchi S, Okuda J, Hamamoto H, Ishii M, Osumi W, Yamamoto M, Inoue Y, Tanaka K, Uchiyama K (2019). Intracorporeal versus extracorporeal anastomosis in laparoscopic left colectomy for left-side colon cancer: a retrospective study. Clin Surg.

[10] Swaid F, Sroka G, Madi H, Shteinberg D, Somri M, Matter I (2016). Totally laparoscopic versus laparoscopic-assisted left colectomy for cancer: a retrospective review. Surg Endosc.

[11] Hamamoto H, Okuda J, Izuhara K, Ishii M, Osumi W, Masubuchi S, Yamamoto M, Tanaka K, Uchiyama K (2021). Closure of enterotomy after side-to-side ileocolic anastomosis with two barbed sutures in totally laparoscopic colectomy for right-sided colon cancer. Surg Today.

[12] Fleshman J, Sargent DJ, Green E, Anvari M, Stryker SJ, Beart RW, Hellinger M, Flanagan R, Peters W, Nelson H (2007). Laparoscopic colectomy for cancer is not inferior to open surgery based on 5-year data from the COST Study Group trial. Ann Surg.

[13] Jayne DG, Guillou PJ, Thorpe H, Quirke P, Copeland J, Smith AM, Heath RM, Brown JM (2007). Randomized trial of laparoscopic-assisted resection of colorectal carcinoma: 3-year results of the UK MRC CLASICC Trial Group. J Clin Oncol.

[14] Lacy AM, Delgado S, Castells A, Prins HA, Arroyo V, Ibarzabal A, Pique JM (2008). The long-term results of a randomized clinical trial of laparoscopy-assisted versus open surgery for colon cancer. Ann Surg.

[15] Jayne DG, Thorpe HC, Copeland J, Quirke P, Brown JM, Guillou PJ (2010). Five-year follow-up of the Medical Research Council CLASICC trial of laparoscopically assisted versus open surgery for colorectal cancer. Br J Surg.

[16] Morikawa E, Yasutomi M, Shindou K, Matsuda T, Mori N, Hida J, Kubo R, Kitao M, Nakamura M, Fujimoto K (1994). Distribution of metastatic lymph nodes in colorectal cancer by the modified clearing method. Dis Colon Rectum.

[17] Toyota S, Ohta H, Anazawa S (1995). Rationale for extent of lymph node dissection for right colon cancer. Dis Colon Rectum.

[18] Mujagic E, Marti WR, Coslovsky M, Soysal SD, Mechera R, von Strauss M, Zeindler J, Saxer F, Mueller A, Fux CA (2018). Associations of hospital length of stay with surgical site infections. World J Surg.

[19] Shapiro R, Keler U, Segev L, Sarna S, Hatib K, Hazzan D (2016). Laparoscopic right hemicolectomy with intracorporeal anastomosis: short- and long-term benefits in comparison with extracorporeal anastomosis. Surg Endosc.

[20] Biondi A, Santocchi P, Pennestrì F, Santullo F, D'Ugo D, Persiani R (2017). Totally laparoscopic right colectomy versus laparoscopically assisted right colectomy: a propensity score analysis. Surg Endosc.

[21] Martinek L, You K, Giuratrabocchetta S, Gachabayov M, Lee K, Bergamaschi R (2018). Does laparoscopic intracorporeal ileocolic anastomosis decreases surgical site infection rate? A propensity score-matched cohort study. Int J Colorectal Dis.

[22] Masubuchi S, Okuda J, Yamamoto M, Inoue Y, Tanaka K, Uchiyama K (2021). Natural orifice specimen extraction in laparoscopic colorectal cancer surgery: a case series study. Int J Surg Case Rep.

[23] Weiss JM, Pfau PR, O'Connor ES, King J, LoConte N, Kennedy G, Smith MA (2011). Mortality by stage for right-versus left-sided colon cancer: analysis of surveillance, epidemiology, and end results—medicare data. J Clin Oncol.

[24] Gervaz P, Usel M, Rapiti E, Chappuis P, Neyroud-Kaspar I, Bouchardy C (2016). Right colon cancer: left behind. Eur J Surg Oncol.

[25] Gray R, Barnwell J, McConkey C, Hills RK, Williams NS, Kerr DJ (2007). Adjuvant chemotherapy versus observation in patients with colorectal cancer: a randomised study. Lancet.

[26] André T, Boni C, Navarro M, Tabernero J, Hickish T, Topham C, Bonetti A, Clingan P, Bridgewater J, Rivera F (2009). Improved overall survival with oxaliplatin, fluorouracil, and leucovorin as adjuvant treatment in stage II or III colon cancer in the MOSAIC trial. J Clin Oncol.

[27] National Comprehensive Cancer Network guidelines. https://www.nccn.org/professionals/physician_gls/pdf/colon.pdf. Accessed 20 Jan 2022.

[28] Allaix ME, Degiuli M, Bonino MA, Arezzo A, Mistrangelo M, Passera R, Morino M (2019). Intracorporeal or extracorporeal ileocolic anastomosis after laparoscopic right colectomy: a double-blinded randomized controlled trial. Ann Surg.

[29] Bollo J, Turrado V, Rabal A, Carrillo E, Gich I, Martinez MC, Hernandez P, Targarona E (2020). Randomized clinical trial of intracorporeal versus extracorporeal anastomosis in laparoscopic right colectomy (IEA trial). Br J Surg.

[30] Serra-Aracil X, Pascua-Solé M, Mora-López L, Vallverdú H, Serracant A, Espina B, Ruiz C, Merichal M, Sánchez A, Romagnolo L (2020). Multicenter controlled study of intracorporeal mechanical side-to-side isoperistaltic anastomosis versus extracorporeal anastomosis in laparoscopic right hemicolectomy: HEMI-D-TREND-Study. Dig Surg.

